# Association between physical activity levels and motoric cognitive risk syndrome in Chinese older adults

**DOI:** 10.1186/s12877-026-07403-z

**Published:** 2026-03-29

**Authors:** Yiming Han, Lulu Geng, Mengzhao Wang, Yifei Wang, Hongli Wang

**Affiliations:** 1 College of Physical Education and Health, Guangxi Normal University, Guilin, China; 2 Institute of Sports and Sports Science, Karlsruhe Institute of Technology, Karlsruhe, Germany

**Keywords:** Physical activity, Motoric cognitive risk syndrome, Older adults, Dementia prevention

## Abstract

**Objective:**

To investigate the cross-sectional and longitudinal associations between physical activity levels (PAL) and motoric cognitive risk (MCR) syndrome in Chinese older adults.

**Methods:**

This study analyzed data from the China Health and Retirement Longitudinal Study (CHARLS) spanning 2011–2015. Cross-sectional analysis included 1,721 participants and longitudinal analysis included 1,506 participants aged ≥ 60 years (mean follow-up: 3.68 years). PAL was quantified using metabolic equivalent of task (MET-minutes/week) and categorized as low (< 600), moderate (600-2,999), or high (≥ 3,000). MCR was defined by concurrent slow gait speed (> 1 SD below age- and sex-specific means) and subjective cognitive complaints. Multivariable logistic regression and Cox proportional hazards models were employed to examine association between PAL and MCR, with restricted cubic spline (RCS) analysis exploring dose-response relationships.

**Results:**

Multivariate logistic regression analysis revealed that moderate and high PAL significantly reduced MCR risks compared with low PAL (moderate: OR = 0.47, 95% CI: 0.28–0.81, *p* = 0.006; high: OR = 0.62, 95% CI: 0.45–0.85, *p* = 0.003). Cox proportional hazards model demonstrated that high PAL was associated with a significantly lower hazard of incident MCR (HR = 0.55, 95% CI: 0.35–0.86, *p* = 0.009). RCS analysis identified a significant nonlinear dose-response relationship (*p* = 0.032), with optimal protective effects observed at 4,133 − 15,000 MET-minutes/week. Subgroup analyses showed particularly robust protective effects in individuals aged < 65 years, married individuals, those with depression, and chronic obstructive pulmonary disease (COPD) patients.

**Conclusion:**

Higher physical activity levels are independently associated with reduced MCR risks and incidence in Chinese older adults, exhibiting a nonlinear dose-response pattern. These findings support physical activity promotion as an accessible, cost-effective preventive strategy for MCR and subsequent dementia risk reduction in aging populations.

## Introduction

With the acceleration of global aging, the prevalence of neurodegenerative diseases such as Alzheimer’s disease has increased substantially, posing a significant global public health challenge. China has entered an aging society, with the number of dementia cases ranking among the highest globally, placing immense pressure on the public healthcare system [1]. According to recent epidemiological data, approximately 57.4 million individuals worldwide are affected by dementias, and this figure is projected to rise continuously as population aging intensifies [2]. Although pharmacological treatments can delay disease progression to some extent, effective therapeutic interventions for dementia remain elusive. Consequently, early identification and intervention for dementia are particularly crucial, especially during the pre-dementia stage [3]. Early interventions can decelerate cognitive decline, improve quality of life, and reduce dementia incidence, thereby alleviating healthcare and societal burdens.

Among pre-dementia conditions, mild cognitive impairment (MCI) is the most widely studied, typically identified through formal neuropsychological testing [4]. However, MCI assessment requires specialized cognitive evaluations that may not be readily accessible in community or primary care settings. Motoric cognitive risk syndrome (MCR), as an alternative pre-dementia construct, is defined by the concurrent presence of subjective cognitive complaints (SCC) and slow gait speed, without requiring neuropsychological testing [5, 6]. This operational simplicity makes MCR particularly advantageous for large-scale population-based screening and epidemiological research. MCR affects approximately 10% of older adults globally and approximately 12% in China, and is strongly associated with elevated risks of dementia, frailty, and falls [5–9]. Notably, slow gait speed—a cardinal feature of MCR—reflects early neuropathological changes including white matter lesions and brain atrophy [10, 11], further supporting MCR as a clinically meaningful pre-dementia marker.

The development of MCR is thought to involve multiple biological mechanisms, including neuroinflammation, oxidative stress, and cytokine imbalance, which may contribute to neuronal damage and reduced neuroplasticity [5, 12]. Additionally, MCR may be influenced by genetic susceptibility, environmental exposures, and modifiable lifestyle factors such as physical inactivity and social isolation [5]. Among these, increasing physical activity levels (PAL) has emerged as a particularly promising modifiable target for MCR prevention.

Currently, prevention and improvement strategies for MCR primarily include behavioral interventions, pharmacological treatments, and lifestyle modifications [5]. Among these approaches, exercise intervention is widely recognized as a critical modality for improving MCR [13]. Research demonstrates that physical activity, particularly aerobic exercise and resistance training, exhibits significant efficacy in improving gait speed and cognitive function [14, 15]. Evidence suggests that physical activity, particularly aerobic exercise (such as brisk walking, cycling, and swimming) and resistance training, demonstrates significant efficacy in improving gait speed and cognitive function [16, 17]. Additionally, aerobic exercise can improve cognitive processing speed, attention, and executive function, playing a vital role in delaying cognitive decline [18]. Resistance training, as another important exercise intervention modality, primarily enhances gait speed and reduces motor impairments by increasing muscle strength, improving motor coordination, and enhancing physical functionality [19]. Resistance training helps improve activities of daily living in older adults, reduce falls and frailty incidence, while also exerting positive effects on brain health. Research further indicates that comprehensive exercise programs combining aerobic exercise and resistance training can simultaneously enhance physical capacity and promote cognitive function improvement, demonstrating significant clinical efficacy [17].

Beyond exercise, multifaceted intervention strategies including adequate sleep, balanced nutrition, social activities, and cognitive training are also considered important approaches for MCR prevention and improvement [9]. Nevertheless, exercise intervention remains the most direct, readily implementable, and cost-effective strategy. Substantial research demonstrates that moderate to vigorous physical activity is closely associated with cognitive function improvement in older adults, particularly in enhancing cognitive function, reducing cognitive decline, and strengthening independent living capacity [20].

Although existing evidence indicates that moderate to high levels of physical activity exert positive effects on the two key domains of MCR (gait speed and cognitive function), the specific dose-response relationship between PAL and MCR remains unclear. Currently, the impact of PAL on MCR in older adults has not been comprehensively evaluated. While some studies support the positive effects of physical activity on MCR, specific dose-response studies examining different intensities, frequencies, and durations of physical activity on MCR incidence and progression are lacking [21].

Therefore, this study utilized data from the China Health and Retirement Longitudinal Study (CHARLS) to investigate the associations between PAL and MCR using three complementary analytical approaches. Multivariable logistic regression was first employed to examine cross-sectional associations between categorized PAL and prevalent MCR at baseline. Because MCR is an incident condition that develops over time and participants may differ in follow-up duration, Cox proportional hazards models were used to evaluate the prospective association between baseline PAL and new-onset MCR, appropriately accounting for variable follow-up time and censoring. Furthermore, given that the dose–response relationship between physical activity and MCR risk may not follow a simple linear pattern—for instance, protective effects may plateau or diminish beyond a certain activity threshold—restricted cubic spline (RCS) analysis was applied to flexibly model this continuous relationship without imposing linearity assumptions. Together, these approaches aim to provide robust evidence for understanding how physical activity may protect against MCR, and to inform the development of evidence-based physical activity recommendations for older adults. 

## Methods

### Study design and population

This study utilized data from the CHARLS, a nationally representative longitudinal cohort study designed to investigate health, socioeconomic status, and family-level factors in Chinese older adults, with the baseline survey conducted during 2011–2012 and biennial follow-up assessments completed in 2013 and 2015 [22]. CHARLS employed a stratified, multistage probability sampling design, conducting face-to-face interviews with individuals aged 45 years and older using structured questionnaires to collect high-quality health-related data. Since the baseline survey in 2011–2012, participants have been followed up biennially. The study was approved by the Institutional Review Board of Peking University (approval number: IRB00001052-11015), and all participants provided informed consent. All procedures involving human participants adhered to the ethical standards of the 1964 Declaration of Helsinki.

**Fig. 1:**
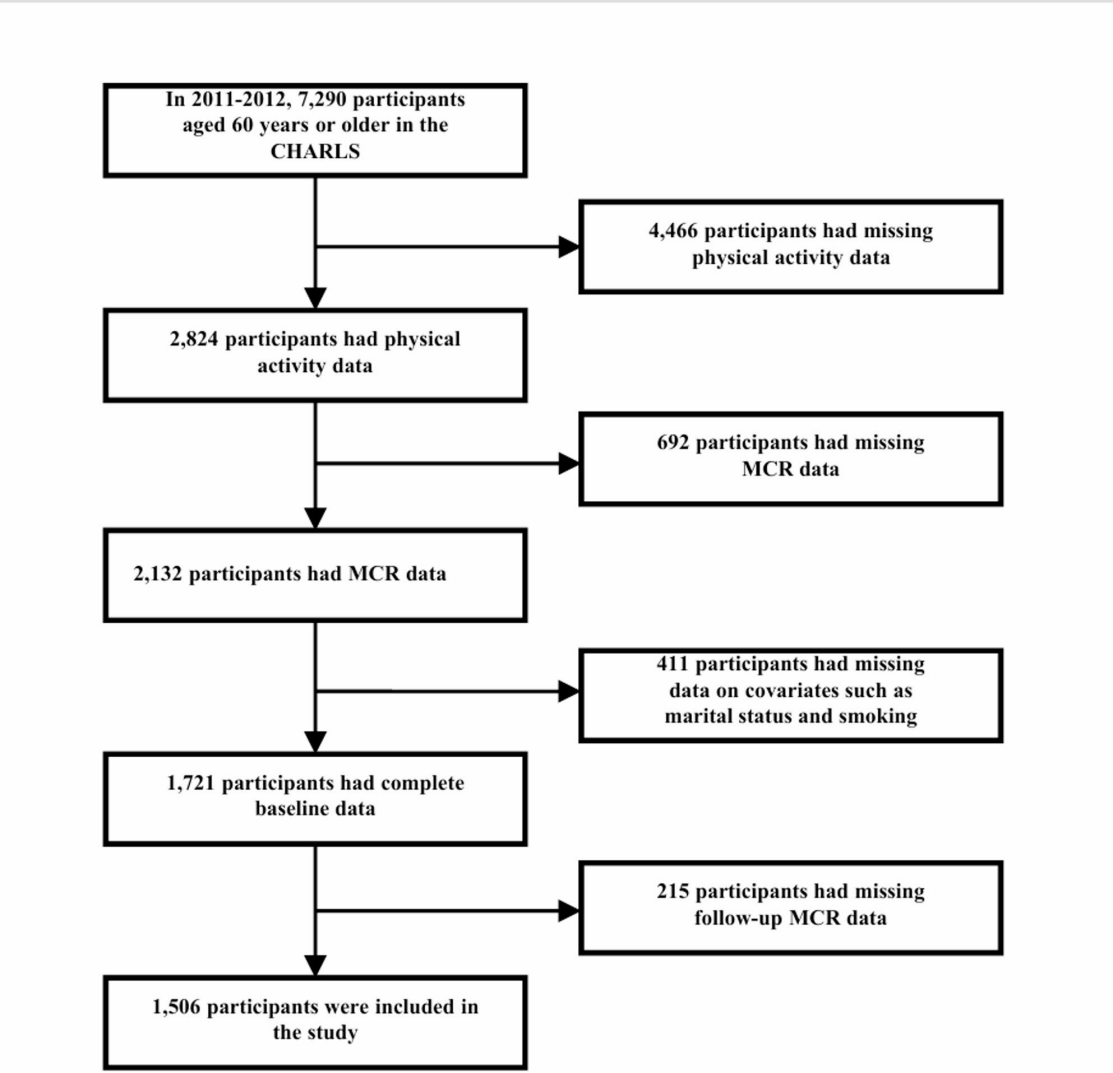
Flow chart of study participant selection

This study analyzed CHARLS data from 2011 to 2015. Fig. 1 shows the selection process of the study population. At baseline, 17,708 participants were enrolled. Participants were excluded based on the following criteria: (1) age < 60 years (*N* = 10,418); (2) missing physical activity data (*N* = 4,466); (3) missing MCR data (*N* = 692); (4) missing covariate data on marital status, smoking, alcohol consumption, hypertension, diabetes, heart disease, chronic obstructive pulmonary disease (COPD), and arthritis (*N* = 411); (4) missing MCR data during follow-up (*N* = 215). Ultimately, 1,721 eligible participants were included in the cross-sectional analysis, and 1,506 eligible participants were included in the longitudinal analysis.

### Physical activity level

PAL was assessed using metabolic equivalent of task (MET) values derived from physical activity data collected via questions adapted from the International Physical Activity Questionnaire (IPAQ), a widely validated instrument for population-level physical activity assessment [23]. Participants self-reported the frequency (days per week) and duration of vigorous-intensity physical activity, moderate-intensity physical activity, and walking performed during a typical week. Duration was recorded using categorical response options (> 4 h, 2–4 h, 30 min to 2 h, and < 30 min), which were converted to midpoint minute values of 240, 180, 75, and 20 min, respectively, for calculation purposes. MET-minutes/week were subsequently computed using established IPAQ MET coefficients: vigorous-intensity activity = 8.0 METs, moderate-intensity activity = 4.0 METs, and walking = 3.3 METs, consistent with previously published CHARLS-based analyses [24, 25]. Total PAL was primarily categorized into three groups: low (< 600 MET-minutes/week), moderate (≥ 600 and < 3,000 MET-minutes/week), and high (≥ 3,000 MET-minutes/week). A combined moderate-to-high PAL category (≥ 600 MET-minutes/week) was additionally constructed for supplementary analyses to increase statistical power, given the relatively small sample size of the moderate PAL group alone. PAL was further classified as a binary variable, with “No PA” defined as MET-minutes/week = 0 (i.e., participants reporting no vigorous-intensity, moderate-intensity, or walking activity) and “Engaging in PA” defined as MET-minutes/week > 0. In addition, PAL was divided into quartiles based on the sample distribution of MET-minutes/week to enable quartile-based analyses.

### MCR assessment

MCR diagnosis was defined as the concurrent presence of slow gait speed and SCC in individuals without dementia or significant mobility disability (inability to walk independently or requiring assistive devices) [26]. Slow gait was defined as walking speed (m/s) more than one standard deviation below the age- specific and sex-specific population mean. The critical values for slow gait speed are as follows: Men (< 75 years) = 0.44 m/s, Men (≥ 75 years) = 0.35 m/s, Women (< 75 years) = 0.41 m/s, Women (≥ 75 years) = 0.33 m/s [27, 28]. SCC assessment was based on participants’ responses to standardized questionnaire items requiring them to rate their current memory or compare it with their previous assessment; responses of “fair” or “poor” were considered indicative of SCC [29, 30].

### Covariates

Covariates were selected for this study based on previous research and the associations between physical activity and MCR, including demographic factors (sex and age), socioeconomic factors (education), living conditions (marital status), behavioral habits (smoking, alcohol consumption), and chronic diseases (depression, hypertension, diabetes, COPD, heart disease, and arthritis). In this study, participants were divided into two age groups: 60–64 years and ≥ 65 years. Notably, continuous age variables were used as covariates in the models, while categorical age variables were used only for age-stratified subgroup analyses. According to the 1997 International Standard Classification of Education (ISCED), education was categorized into three levels: below high school, high school, and above high school. Marital status was classified as married/partnered or unmarried/other, with the unmarried/other category including separated, divorced, widowed, or never married individuals. Living with children was categorized as: no or yes. Smoking behavior was classified into two categories: currently non-smoking or currently smoking. Alcohol consumption was assessed based on the number of alcoholic drinks in the past week and classified as: non-excessive drinking or excessive drinking. Depression was categorized as present or absent, with Center for Epidemiologic Studies Depression Scale (CES-D) scores < 10 indicating absence and scores ≥ 10 indicating presence of depression. Hypertension, diabetes, COPD, heart disease, and arthritis diagnoses were all ascertained through self-reported physician diagnosis.

### Statistical analysis

Continuous variables were expressed as mean ± standard deviation (SD), and categorical variables as numbers (percentages). Multivariable logistic regression models were employed to analyze associations between PAL (low, moderate, high) and PAL quartiles with MCR. Models were progressively adjusted to evaluate the independent effects of physical activity. Model 1 was unadjusted. Model 2 was adjusted for age and sex. Model 3 was further adjusted for marital status, education level, smoking status, alcohol consumption status, depression, hypertension, diabetes, COPD, heart disease, and arthritis. Results were reported using odds ratios (OR), 95% confidence intervals (CI), and P values. Using the low activity group as reference, the effects of moderate and high activity levels on individual MCR risk were evaluated.

This study employed Cox proportional hazards models to analyze associations between PAL (low, moderate, high) and PAL quartiles with MCR incidence, with progressive model adjustments to evaluate the independent effects of physical activity. RCS analysis was conducted within the Cox proportional hazards framework, modeling the log hazard as a smooth function of continuous MET-minutes/week, with knots placed at the 10th, 50th, and 90th percentiles following established recommendations. This analysis served three complementary purposes: to formally test for departure from linearity in the dose–response relationship between MET-minutes/week and MCR risk (with the p-value for nonlinearity reported); to identify potential threshold values or inflection points at which the protective association of physical activity plateaus or attenuates, thereby informing clinically meaningful dose recommendations; and to complement the categorical analyses (low/moderate/high PAL and quartile-based groupings) by providing a continuous, assumption-free visualization of the exposure–outcome relationship, avoiding the information loss inherent in categorization of a continuous exposure variable. Subgroup analyses were conducted for variables including age group(aged < 65 years vs. aged ≥ 65 years), sex, marital status, education level, smoking status, alcohol consumption status, depression, hypertension, diabetes, COPD, heart disease, and arthritis. All analyses were performed using STATA (version 17.0) and R (version 4.3.2), with two-sided p values < 0.05 considered statistically significant.

## Results

### Baseline characteristics

A total of 1,721 eligible participants were included at baseline, with a mean age of 66.38 years and 53% female. Table 1 presents cross-sectional baseline characteristics of the study population by PAL. Participants in the low PAL group were older, had a higher proportion of females, lower smoking prevalence, and higher prevalence of hypertension, depression, and MCR. At follow-up baseline, 1,506 eligible participants were included, with a mean follow-up duration of 3.68 years. Table 2 presents longitudinal (follow-up cohort) baseline characteristics of the study population by PAL. Participants in the low PAL group were older and had higher proportions of females and hypertension patients.

**Table 1:** Cross-sectional demographic characteristics of participants stratified by baseline PAL

Variables	Total (*n* = 1721)	PAL			*P*
		Low *n* = 577	Moderate *n* = 217	High *n* = 927	
Age, mean (SD), years	66.38 (6.66)	67.90 (7.08)	66.32 (6.82)	65.44 (6.16)	< 0.001
Sex					< 0.001
Male	809 (47%)	225 (39%)	101 (47%)	483 (52%)	
Female	912 (53%)	352 (61%)	116 (53%)	444 (48%)	
Marital status					< 0.001
No	334 (19%)	148 (26%)	44 (20%)	142 (15%)	
Yes	1,387 (81%)	429 (74%)	173 (80%)	785 (85%)	
Education level					0.034
Low education level	1,645 (96%)	547 (95%)	201 (93%)	897 (97%)	
Secondary education level	56 (3.3%)	24 (4.2%)	12 (5.5%)	20 (2.2%)	
High education level	20 (1.2%)	6 (1.0%)	4 (1.8%)	10 (1.1%)	
Smoking status					0.006
No	1,055 (61%)	383 (66%)	133 (61%)	539 (58%)	
Yes	666 (39%)	194 (34%)	84 (39%)	388 (42%)	
Drinking alcohol					0.010
No	1,433 (83%)	500 (87%)	184 (85%)	749 (81%)	
Yes	288 (17%)	77 (13%)	33 (15%)	178 (19%)	
Depression					0.043
No	1,024 (60%)	335 (58%)	146 (67%)	543 (59%)	
Yes	697 (40%)	242 (42%)	71 (33%)	384 (41%)	
Hypertension					< 0.001
No	1,162 (68%)	355 (62%)	134 (62%)	673 (73%)	
Yes	559 (32%)	222 (38%)	83 (38%)	254 (27%)	
Diabetes					0.083
No	1,594 (93%)	523 (91%)	203 (94%)	868 (94%)	
Yes	127 (7.4%)	54 (9.4%)	14 (6.5%)	59 (6.4%)	
COPD					0.3
No	1,490 (87%)	497 (86%)	182 (84%)	811 (87%)	
Yes	231 (13%)	80 (14%)	35 (16%)	116 (13%)	
Heart disease					< 0.001
No	1,450 (84%)	466 (81%)	163 (75%)	821 (89%)	
Yes	271 (16%)	111 (19%)	54 (25%)	106 (11%)	
Arthritis					0.4
No	1,098 (64%)	371 (64%)	130 (60%)	597 (64%)	
Yes	623 (36%)	206 (36%)	87 (40%)	330 (36%)	
MCR					< 0.001
No	1,506 (88%)	477 (83%)	199 (92%)	830 (90%)	
Yes	215 (12%)	100 (17%)	18 (8.3%)	97 (10%)	

*PAL*  Physical activity level, *MCR*  Motoric cognitive risk syndrome, *COPD*  Chronic obstructive pulmonary disease, *SD*  Standard deviation, *MET*  Metabolic equivalent of task

chi-square test for categorical variables and analysis of variance for continuous variables

**Table 2:** Longitudinal follow-up demographic characteristics of participants stratified by baseline PAL

Variables	Total (*n* = 1506)	PAL			*P*
		Low *n* = 477	Moderate *n* = 199	High *n* = 830	
Age, mean (SD), years	66.22 (6.70)	67.71 (7.04)	66.15 (6.87)	65.38 (6.32)	< 0.001
Sex					< 0.001
Male	720 (48%)	189 (40%)	96 (48%)	435 (52%)	
Female	786 (52%)	288 (60%)	103 (52%)	395 (48%)	
Marital status					0.005
No	274 (18%)	108 (23%)	38 (19%)	128 (15%)	
Yes	1,232 (82%)	369 (77%)	161 (81%)	702 (85%)	
Education level					0.032
Low education level	1,435 (95%)	450 (94%)	183 (92%)	802 (97%)	
Secondary education level	52 (3.5%)	21 (4.4%)	12 (6.0%)	19 (2.3%)	
High education level	19 (1.3%)	6 (1.3%)	4 (2.0%)	9 (1.1%)	
Smoking status					0.020
No	920 (61%)	314 (66%)	124 (62%)	482 (58%)	
Yes	586 (39%)	163 (34%)	75 (38%)	348 (42%)	
Drinking alcohol					0.051
No	1,250 (83%)	411 (86%)	167 (84%)	672 (81%)	
Yes	256 (17%)	66 (14%)	32 (16%)	158 (19%)	
Depression					0.041
No	922 (61%)	285 (60%)	138 (69%)	499 (60%)	
Yes	584 (39%)	192 (40%)	61 (31%)	331 (40%)	
Hypertension					< 0.001
No	1,036 (69%)	299 (63%)	127 (64%)	610 (73%)	
Yes	470 (31%)	178 (37%)	72 (36%)	220 (27%)	
Diabetes					0.065
No	1,401 (93%)	433 (91%)	187 (94%)	781 (94%)	
Yes	105 (7.0%)	44 (9.2%)	12 (6.0%)	49 (5.9%)	
COPD					0.3
No	1,300 (86%)	411 (86%)	165 (83%)	724 (87%)	
Yes	206 (14%)	66 (14%)	34 (17%)	106 (13%)	
Heart disease					< 0.001
No	1,269 (84%)	385 (81%)	149 (75%)	735 (89%)	
Yes	237 (16%)	92 (19%)	50 (25%)	95 (11%)	
Arthritis					0.5
No	976 (65%)	316 (66%)	122 (61%)	538 (65%)	
Yes	530 (35%)	161 (34%)	77 (39%)	292 (35%)	

*PAL*  Physical activity level, *MCR*  Motoric cognitive risk syndrome, *COPD*  Chronic obstructive pulmonary disease, *SD*  Standard deviation, *MET*  Metabolic equivalent of task

chi-square test for categorical variables and analysis of variance for continuous variables

### Association between PAL and baseline MCR

Table 3 shows the association between PAL and baseline MCR. In logistic regression analyses of low, moderate, and high PAL stratified by MET, compared with low PAL, both moderate and high PAL in all three models were associated with progressively decreased MCR risk (moderate PAL Model 3: OR = 0.47, 95% CI: 0.28–0.81, *p* = 0.006; high PAL Model 3: OR = 0.62, 95% CI: 0.45–0.85, *p* = 0.003; Moderate-to-High PAL Model 3: OR = 0.59, 95% CI: 0.43–0.80, *p* < 0.001). In logistic regression analyses of PAL quartiles stratified by MET, compared with Q1, only Q4 in all three models was associated with progressively decreased MCR risk (Model 3: OR = 0.62, 95% CI: 0.41–0.94, *p* = 0.026). Although Q2 and Q3 in all three models showed some reduction in MCR risk, neither reached statistical significance.

**Table 3:** Multivariate logistic regression analysis of the association between PAL and MCR

Variables	Model 1		Model 2		Model 3	
	OR (95%Cl)	*P*	OR (95%Cl)	*P*	OR (95%Cl)	*P*
PAL						
Low	1.00(Ref)		1.00(Ref)		1.00(Ref)	
Moderate	0.43 (0.25–0.73)	0.002	0.45 (0.27–0.77)	0.003	0.47 (0.28–0.81)	0.006
High	0.56 (0.41–0.75)	< 0.001	0.60 (0.44–0.82)	0.001	0.62(0.45–0.85)	0.003
Moderate-to-High	0.53 (0.40–0.71)	< 0.001	0.57 (0.43–0.77)	< 0.001	0.59 (0.43–0.80)	< 0.001
PAL quartile						
Q1	1.00(Ref)		1.00(Ref)		1.00(Ref)	
Q2	0.80 (0.54–1.18)	0.260	0.82 (0.56–1.22)	0.333	0.83 (0.56–1.24)	0.372
Q3	0.72 (0.49–1.06)	0.092	0.76 (0.52–1.13)	0.175	0.78 (0.52–1.15)	0.211
Q4	0.55 (0.37–0.83)	0.004	0.62 (0.40–0.94)	0.023	0.62 (0.41–0.94)	0.026

*PAL*  Physical activity level

Model 1 is unadjusted, Model 2 is adjusted for age and sex, and Model 3 further adjusts for marital status, education level, smoking, alcohol consumption, depression, hypertension, diabetes, COPD, heart disease, and arthritis.

The corresponding MET ranges for each PAL quartile are as follows: Q1: 0-198; Q2: 240–4,158; Q3: 4,200–10,584; Q4: 10,640–25,704

### Association between PAL and the risk of MCR occurrence

Table 4 shows the association between PAL and the risk of MCR occurrence. In Cox proportional hazards models of low, moderate, and high PAL stratified by MET, compared with low PAL, high PAL in all three models was associated with progressively decreased MCR risk (Model 3: HR = 0.55, 95% CI: 0.35–0.86, *p* = 0.009). Compared with low PAL, moderate PAL in all three models showed some reduction in MCR risk but did not reach statistical significance (Model 3: HR = 0.61, 95% CI: 0.31–1.20, *p* = 0.155). However, compared with low PAL, Moderate-to-High PAL in all three models was associated with progressively decreased MCR risk (Model 3: HR = 0.56, 95% CI: 0.37–0.86, *p* = 0.008). Compared with no PA, engaging in PA in all three models was associated with progressively decreased MCR risk (Model 3: HR = 0.44, 95% CI: 0.29–0.67, *p* < 0.001). In Cox proportional hazards models of PAL quartiles stratified by MET, compared with Q1, Q2, Q3, and Q4 in all three models were associated with progressively decreased MCR risk (Q2 Model 3: HR = 0.43, 95% CI: 0.24–0.78, *p* = 0.005; Q3 Model 3: HR = 0.44, 95% CI: 0.25–0.77, *p* = 0.004; Q4 Model 3: HR = 0.44, 95% CI: 0.25–0.77, *p* = 0.004).

**Table 4:** Association between baseline PAL and the risk of MCR occurrence

Variables	Model 1		Model 2		Model 3	
	HR (95%Cl)	*P*	HR (95%Cl)	*P*	HR (95%Cl)	*P*
PAL						
Low	1.00(Ref)		1.00(Ref)		1.00(Ref)	
Moderate	0.55 (0.28–1.07)	0.078	0.58 (0.30–1.14)	0.114	0.61 (0.31–1.20)	0.155
High	0.53 (0.34–0.81)	0.003	0.58 (0.37–0.90)	0.014	0.55 (0.35–0.86)	0.009
Moderate-to-High	0.53 (0.35–0.80)	0.002	0.58 (0.38–0.88)	0.010	0.56 (0.37–0.86)	0.008
No PA	1.00(Ref)		1.00(Ref)		1.00(Ref)	
Engaging in PA	0.41 (0.27–0.62)	< 0.001	0.44 (0.29–0.67)	< 0.001	0.44 (0.29–0.67)	< 0.001
PAL quartile						
Q1	1.00(Ref)		1.00(Ref)		1.00(Ref)	
Q2	0.40 (0.22–0.72)	0.002	0.41 (0.23–0.74)	0.003	0.43 (0.24–0.78)	0.005
Q3	0.41 (0.24–0.71)	0.002	0.44 (0.25–0.77)	0.004	0.44 (0.25–0.77)	0.004
Q4	0.42 (0.25–0.72)	0.001	0.47 (0.27–0.81)	0.007	0.44 (0.25–0.77)	0.004

*PAL*  Physical activity level

Model 1 is unadjusted, Model 2 is adjusted for age and sex, and Model 3 further adjusts for marital status, education level, smoking, alcohol consumption, depression, hypertension, diabetes, COPD, heart disease, and arthritis. No PA was defined as MET = 0, while engaging in PA was defined as MET > 0

The corresponding MET ranges for each PAL quartile are as follows: Q1: 0–300; Q2: 320–4,158; Q3: 4,200–10,878; Q4: 11,040–25,704

### RCS analysis

**Fig. 2:**
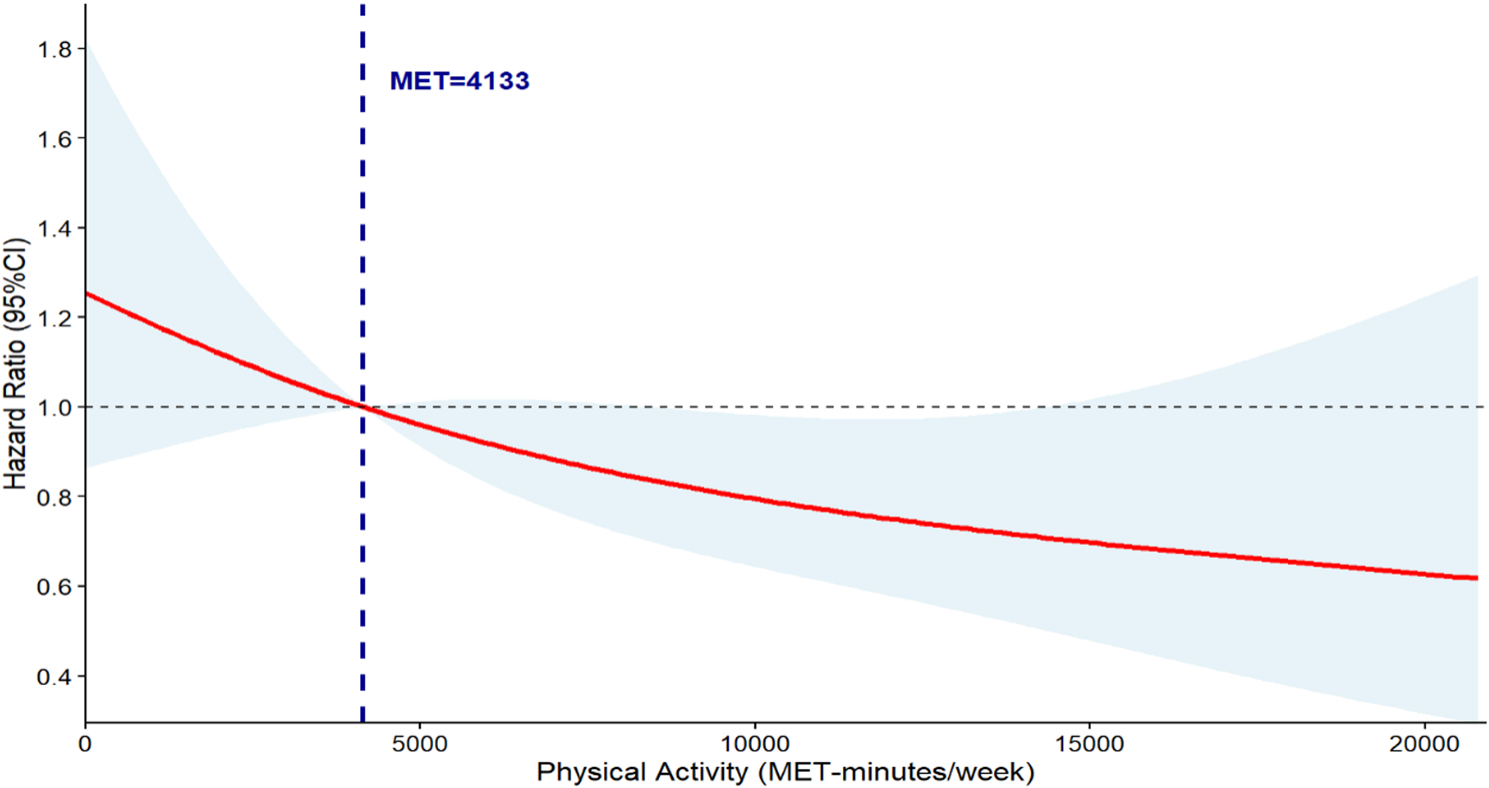
Dose-response relationship between total physical activity (MET-minutes/week) and the risk of MCR occurrence. Note: The model was adjusted for age, sex, marital status, education level, smoking, alcohol consumption, depression, hypertension, diabetes, COPD = chronic obstructive pulmonary disease, heart disease, and arthritis. The figure shows the HR (solid line) and 95% CI (shaded area)

RCS models adjusted for all covariates were employed for analysis. The Fig. 2 demonstrates a significant nonlinear relationship between PAL and MCR incidence (*p* = 0.032). When PAL < 4,133 MET-minutes/week, HR < 1.00. The overall curve trend showed a negative correlation, with HR values decreasing as physical activity volume increased. This indicates that higher levels of physical activity are associated with lower MCR risk. When MET-minutes/week > 15,000, the confidence interval widened and crossed 1, reflecting that the protective effect of physical activity lacks statistical significance when PAL exceeds 15,000 MET-minutes/week.

### Subgroup analysis

**Fig. 3:**
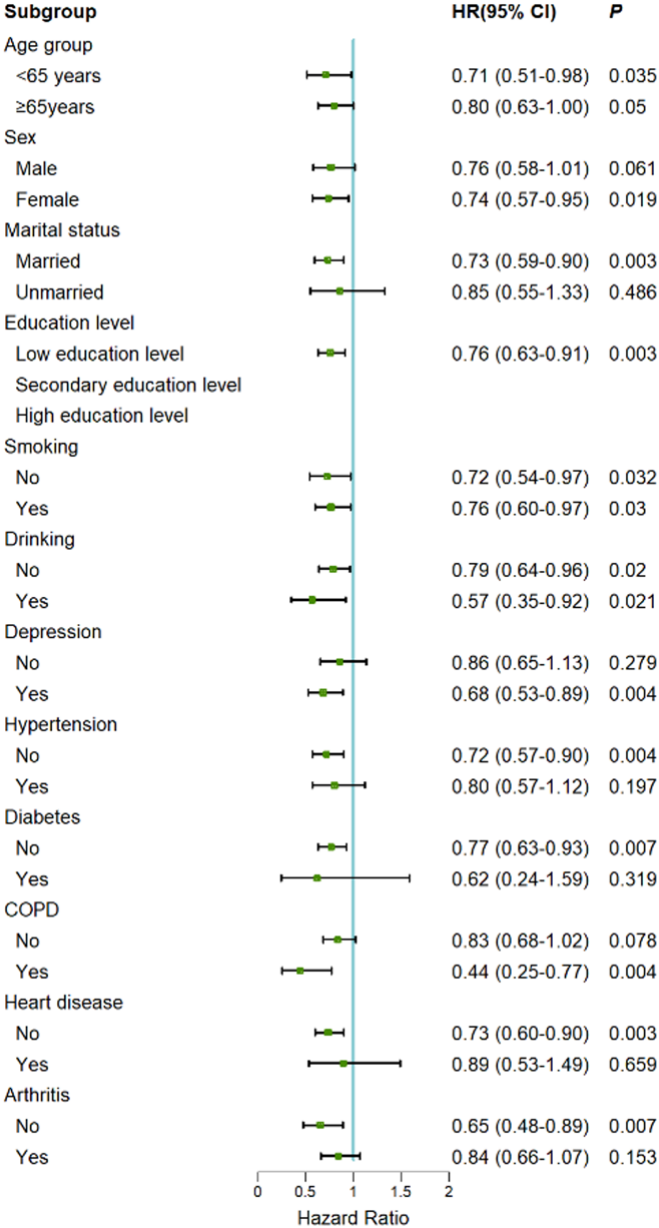
Association between baseline PAL and the occurrence of incident MCR subgroups. Note: Subgroup analysis was conducted using the Cox proportional hazards model (Model 3). Age was divided into two groups (60–64 years and ≥ 65 years). The model was adjusted for age, sex, marital status, education level, smoking, alcohol consumption, depression, hypertension, diabetes, COPD = chronic obstructive pulmonary disease, heart disease, and arthritis. Each square represents the HR for a specific group, and the horizontal line through the square indicates the 95% CI. The vertical dashed line represents the null value of HR = 1.00

The subgroup analysis results in Fig. 3 show that physical activity has a significant protective effect in the following groups: age < 65 years (HR = 0.71, *P* = 0.035), married (HR = 0.73, *P* = 0.003), depressed (HR = 0.68, *P* = 0.004), and those with COPD (HR = 0.44, *P* = 0.004). Physical activity demonstrated robust protective effects regardless of smoking and alcohol consumption levels. Additionally, physical activity exhibited protective effects in populations without diabetes, heart disease, and arthritis. Subgroup analysis could not be performed for education level strata due to excessively small participant numbers and absence of MCR events in the moderate education level group (*n* = 43, events = 0) and high education level group (*n* = 17, events = 0).

## Discussion

This study systematically examined cross-sectional and longitudinal associations between PAL and MCR. Results demonstrate that higher levels of physical activity are closely associated with significant reductions in MCR risk, with this association remaining robust after adjusting for multiple potential confounding factors. Cross-sectional analysis revealed that compared with low PAL, moderate PAL was associated with significantly lower odds of prevalent MCR (OR = 0.47, 95% CI: 0.28–0.81), as was high PAL (OR = 0.62, 95% CI: 0.45–0.85). Longitudinal analysis further confirmed that high PAL was associated with a significantly lower hazard of incident MCR (HR = 0.55, 95% CI: 0.35–0.86). Moderate PAL alone did not reach statistical significance in the longitudinal analysis (HR = 0.61, 95% CI: 0.31–1.20). Therefore, we combined moderate and high PAL for analysis and found that, compared with low PAL, moderate-to-high PAL was associated with a significantly lower hazard of incident MCR (HR = 0.56, 95% CI: 0.37–0.86). Further stratified analyses using PA as a binary exposure confirmed that, compared with no PA, engaging in any PA was associated with a significantly lower hazard of incident MCR (HR = 0.44, 95% CI: 0.29–0.67). Moreover, quartile stratification of MET indicated that, compared with the first quartile, all other quartiles significantly reduced the risk of MCR incidence, demonstrating that both moderate-to-high PAL and simply engaging in PA can effectively lower the risk of developing MCR.

Previous research indicates that PAL is an important modifiable risk factor for dementia, with moderate to high levels of physical activity being significantly associated with decelerated cognitive decline in middle-aged and older adults. A systematic review and meta-analysis including 49 observational cohort studies with 257,983 participants demonstrated that higher self-reported PAL was associated with significantly lower odds or hazard of any type of dementia, Alzheimer’s disease, or vascular dementia, exhibiting a dose-gradient response [31]. A recent study using the UK Biobank provided more precise dose-response relationship evidence. This study included 89,667 adults with a mean follow-up of 4.4 years, reporting that each additional 30 min of moderate-to-vigorous physical activity was associated with a lower hazard of dementia [32]. Compared with zero minutes of moderate-to-vigorous physical activity per week, participants engaging in 35–69.9 min per week had a lower hazard of dementia (HR = 0.40), with similarly lower hazard observed for 70–139.9 min (HR = 0.37) and ≥ 140 min (HR = 0.31) [32].Previous research has focused on the preventive and therapeutic effects of PAL on dementia. To our knowledge, few studies have specifically examined the longitudinal association and dose–response relationship between quantified PAL and MCR risk. The present study contributes to this limited evidence base by employing both cross-sectional and longitudinal designs with continuous dose–response analysis in a nationally representative Chinese older adult cohort, further confirming that the protective effect of physical activity exhibits a “threshold effect” rather than “more is always better.” Even moderate levels of physical activity can significantly reduce MCR risk. This has important guiding significance for developing realistic and feasible exercise intervention programs for older adult populations. Notably, this study also found that when PAL exceeds 15,000 MET-minutes/week, the statistical significance of the protective effect diminishes. This suggests the existence of an “optimal dose window,” where excessive exercise may not confer additional benefits and may even produce negative effects [33]. This aligns with recognition in the sports medicine field regarding overtraining syndrome and mitochondrial damage, reminding us that individual differences and moderation principles should be considered when recommending physical activity.

MCR, as a preclinical stage of dementia, was first conceptualized by Verghese et al. in 2014. Research demonstrates that MCR patients have 3.27 times the risk of developing dementia compared with normal individuals, with the risk of developing vascular dementia reaching 12.81 times higher [34]. However, large-scale longitudinal studies supporting how physical activity influences MCR incidence and progression, as well as its specific dose-response relationship, have been lacking. Regarding MCR components-gait speed and SCC-substantial research has demonstrated that moderate to high PAL can enhance or maintain physical function in middle-aged and older adults to delay gait speed decline. The life study, including 1,635 sedentary older adults aged 70–89 years, showed that after a mean follow-up of 2.6 years, the quartile with the greatest increase in physical activity had a 77% lower risk of major mobility disability compared with the lowest quartile [35]. Another 8-year cohort study including 439 older adults found that gait speed improvers (improvement ≥ 0.1 m/s) had a mortality rate of 31.6%, while non-improvers had a rate of 49.3% [36]. A larger pooled analysis including 34,485 community-dwelling older adults with 6–21 years of follow-up demonstrated that each 0.1 m/s increase in gait speed reduced mortality risk by 12% [37]. Higher PAL can significantly improve SCC, with regular physical activity exerting protective effects against subjective cognitive complaints and reducing cognitive decline risk. A large-scale study across 47 low- and middle-income countries including 248,504 adults demonstrated that in individuals aged ≥ 65 years, low PAL was significantly associated with higher odds of more severe memory complaints and learning complaints [38]. A US study of adults aged ≥ 45 years found that SCD prevalence decreased progressively across inactive, insufficiently active, and sufficiently active groups (15.7%, 11.4%, and 8.8%, respectively), presenting a significant dose-response pattern [39]. A cross-sectional study including 5,328 community-dwelling older adults demonstrated that engaging in ≥ 150 min per week of moderate-to-vigorous physical activity was associated with lower odds of SCC (OR = 0.85, 95% CI: 0.74–0.97) [40].

Our research findings exhibit certain similarities with previous related studies. For example, a multinational study including 17 countries with over 26,000 older adults showed that global MCR prevalence is approximately 9.7%, with regular moderate-to-vigorous physical activity associated with reduced MCR prevalence [21]. However, that study was primarily based on cross-sectional design and could not establish causal relationships. In contrast, this study employed a longitudinal cohort design, enabling better inference of the preventive effects of physical activity on MCR incidence and providing stronger causal inference evidence. Notably, the MCR prevalence observed in Chinese older adults in this study differs somewhat from international study results. This may be related to unique lifestyle, dietary habits, healthcare accessibility, and cultural background of Chinese older adults.

Several potential biological mechanisms may underlie the observed associations between physical activity and MCR, although these remain speculative as the relevant biomarkers were not directly measured in the present study. One plausible explanation involves brain-derived neurotrophic factor (BDNF), inflammation, and cerebrovascular function [5]. Previous studies have suggested that physical activity may exert neuroprotective effects by increasing BDNF expression. BDNF has been reported to play critical roles in neuroplasticity, neurogenesis, and neuronal protection [41, 42]. Aerobic exercise has been shown to increase BDNF levels and may promote synaptic plasticity and neurogenesis in the hippocampus. Moderate-intensity aerobic exercise (60–70% maximum heart rate) for 30–40 min per session, 3–4 times per week, has been suggested to optimally stimulate BDNF production and hippocampal neurogenesis [43], and BDNF signaling may thereby enhance learning and memory capacity [44]. A second potential pathway involves neuroinflammation. Recent research has found that MCR is closely associated with sarcopenia and systemic inflammation, with MCR patients exhibiting elevated TNF-α levels and decreased IL-10/TNF-α and PRGN/TNF-α ratios [45]. Physical exercise has been hypothesized to decelerate neurological disease progression by reducing oxidative stress and neuroinflammation, including potentially reducing amyloid accumulation [46]. The significant protective effects of physical activity observed in the depression and COPD subgroups in the present study may be partially consistent with this hypothesis, as both conditions are characterized by heightened inflammatory burden. A third plausible mechanism relates to cerebrovascular function. Aerobic exercise may increase heart rate and enhance cerebral blood flow, promoting delivery of essential nutrients and oxygen to the brain. Cardiorespiratory fitness has been proposed to mediate neuroprotective effects through improved cerebral blood flow, reduced inflammation, and enhanced neuroplasticity [47]. Slow gait, as a cardinal manifestation of MCR, has been demonstrated to be closely associated with pathological features including white matter lesions, brain atrophy, and neuronal loss [48]. Based on these converging lines of indirect evidence, we hypothesize that physical activity may delay MCR incidence and progression through mechanisms involving upregulation of BDNF, attenuation of neuroinflammation, and improvement of cerebrovascular function; however, these mechanistic pathways remain hypothetical and require confirmation in future studies incorporating direct biomarker measurements.

From a public health perspective, promoting physical activity as a prevention strategy for MCR and dementia offers multiple advantages: low cost, easy accessibility, minimal side effects, strong sustainability, and simultaneous improvement of multiple health outcomes. The neuroprotective mechanisms of exercise include improving cerebral blood flow, reducing inflammation, and enhancing neuroplasticity; these mechanisms interact synergistically to maintain brain health. However, translating research evidence into effective clinical practice and public health policies remains challenging. Future research should focus on how to improve exercise participation rates and adherence in older adult populations, how to develop personalized exercise programs for older adults with different health conditions and functional levels, and how to combine physical activity interventions with other lifestyle interventions to achieve synergistic effects.

### Strengths and limitations

This study, based on a large-sample, multi-wave nationally representative longitudinal cohort study, provides high-quality epidemiological evidence. Employing standardized MCR diagnostic criteria ensures result comparability and reliability. Quantitative assessment of physical activity through MET calculation methods, comprehensively considering physical activity intensity, duration, and frequency, provides more precise exposure measurement than simple categorization. Using RCS analysis to explore nonlinear dose-response relationships provides more nuanced insights into understanding the complex associations between physical activity and MCR. Extensive subgroup analyses and covariate adjustments enhance the robustness and generalizability of study findings.

This study has several limitations requiring consideration in future research. Physical activity data based on self-reports may be subject to recall bias and social desirability bias, leading to overestimation or underestimation of true PAL. Using objective measurement tools such as accelerometers may provide more accurate physical activity assessment. Although this study conducted extensive covariate adjustment, residual confounding factors may still exist, such as dietary quality and biomarkers that were not adequately measured. Biomarkers including BDNF, inflammatory cytokines, and cerebrovascular parameters were not assessed in the present study; therefore, the mechanistic pathways discussed in relation to physical activity and MCR are hypothetical and require confirmation in future studies incorporating direct biomarker measurements. The relatively small sample sizes in certain subgroups (such as higher education level groups) limit the statistical power of subgroup analyses. The mean follow-up duration of 3.68 years is relatively short and may be insufficient to observe the full spectrum of long-term effects of physical activity on MCR. Due to cohort data limitations, this study could not evaluate the impact of changes in physical activity patterns (such as transitioning from low to high activity) on MCR risk.

## Conclusion

Based on longitudinal cohort data from CHARLS, this study demonstrated that higher PAL was independently associated with reduced MCR prevalence and incidence in Chinese older adults, with a nonlinear dose–response relationship indicating optimal protective effects at 4,133–15,000 MET-minutes/week. These findings support promoting physical activity, even at moderate levels, as an accessible and cost-effective strategy for MCR prevention in aging populations. Future interventional studies incorporating biomarker assessments are warranted to establish causality and elucidate the underlying mechanisms.

## Data Availability

The datasets that support the findings of this study are available on the CHARLS website ([http://charls.pku.edu.cn/en/]).

## Abbreviations

PAL: Physical activity levels
MCR: Motoric cognitive risk
CHARLS: China Health and Retirement Longitudinal Study
MET: Metabolic equivalent
SCC: Subjective cognitive complaints
COPD: Chronic obstructive pulmonary disease
RCS: Restricted cubic spline
BDNF: Brain-derived neurotrophic factor
